# Radiographic Trends for Infield Recurrence After Radiosurgery for Cerebral Metastases

**DOI:** 10.7759/cureus.8680

**Published:** 2020-06-17

**Authors:** Arthur Carminucci, Sabrina Zeller, Shabbar Danish

**Affiliations:** 1 Neurosurgery, Rutgers Robert Wood Johnson Medical School, Piscataway, USA; 2 Neurosurgery, Rutgers Robert Wood Johnson Medical School, New Brunswick, USA

**Keywords:** gamma knife, brain metastases, stereotactic radiosurgery, neuro-oncology, infield recurrence

## Abstract

Objective

Recurrence following stereotactic radiosurgery (SRS) for the treatment of cerebral metastases is not uncommon. Recurrence can represent recurrent tumor and/or radiation necrosis. The radiographic response to Gamma Knife (GK) treatment is variable with some remaining stable, some decreasing in size, some increasing in size, while some may show a combination of all three. For tumors that demonstrate progression on MRI, the question to intervene with additional surgical or radiation therapy and the timing of such intervention remains debatable. In this study, we retrospectively reviewed surveillance MRIs of post-GK cerebral metastases to determine if radiographic trends are a predictor of infield progression.

Methods

A retrospective review of cerebral metastases treated with GK radiosurgery with at least two consecutive post-GK MRI scans was performed. Infield progression was defined by new enhancement increased by at least 25% in two out of three dimensions on two consecutive scans. Primary endpoints for infield recurrence were either continued observation, therapeutic intervention, or withdrawal of care.

Results

A total of 579 cerebral metastases were treated with GK radiosurgery. A total of 123 metastases demonstrated radiographic progression on one follow-up MRI scan. Of those, 75% demonstrated continued progression follow-up imaging, while 25% stabilized or regressed. For post-GK metastases demonstrating progression on two consecutive MRI scans, 85% of lesions continued to progress, whereas only 15% demonstrated stabilization or regression. A total of 91% of lesions either require intervention or demonstrate continued progression with observation at this timepoint. Cumulatively 100% of metastases with radiographic progression on ≥3 consecutive MRIs went on to need further intervention.

Conclusion

Approximately one-fourth of infield recurrence demonstrating progression on the first surveillance MRI will stabilize or regress. Those demonstrating infield progression on two consecutive MRI scans should be considered treatment failures. Early interventions before tumor volume increases in size or patients require high-dose steroids maybe beneficial.

## Introduction

Stereotactic radiosurgery (SRS) is commonly used in the treatment of intracerebral metastases [1]. This technique has been shown to be successful as a sole treatment for cerebral metastases and has been favored over other treatment options due to its tolerability and ability to be performed as an outpatient therapy [2].

The radiographic response to radiosurgery is variable; tumors may remain stable, decrease in size, increase in size, or demonstrate a combination of all three [3]. A subsequent increase in size following treatment can represent recurrent tumor or radiation necrosis [4]. Recurrence following SRS is not uncommon, and has been shown in previous studies to occur in anywhere from 7% to 31% of treated tumors [2,3,5-7]. Recurrence following SRS can represent recurrent tumor, radiation necrosis, or a combination of both [8,9]. Despite advanced radiographic modalities, including MRI, spectroscopy and perfusion-weighted imaging, distinguishing recurrent tumor from radiation necrosis is often difficult from imaging alone. Similarly, biopsies of these lesions can display mixed results, with areas of both recurrent tumor and necrosis on pathological specimens [10]. Treatment for infield progression can include medical management with steroids or Avastin for pure radiation necrosis, or repeat radiosurgery or open surgical intervention for pure recurrent tumor [11-13]. The emergence of laser interstitial thermal therapy (LITT) has been shown to be effective for the treatment of both recurrent tumor and radiation necrosis [14]. The question to intervene with additional surgical or radiation therapy and the timing of such intervention is debatable. Many current treatment paradigms for infield progression are based on the concept that pure radiation necrosis can be managed conservatively with medical treatments and observation will ultimately stabilize or regress, whereas pure recurrent tumor requires surgical or radiosurgical intervention. This rationale may be flawed since radiation necrosis can become irreversibly progressive, requiring surgery, and predicting which patients will demonstrate progression is not possible [15].

Given the difficulty in distinguishing radiation necrosis from recurrent tumor, and controversies in management, we aimed to understand the behavior of potentially progressive infield recurrences. Specifically, can trends in radiographic progression on MRI predict the need for intervention for infield recurrence? Studies have not yet been completed to investigate the necessity and timing of intervention following radiographic evidence of recurrence. In this study, we retrospectively reviewed surveillance MRIs of post-SRS cerebral metastases to determine if radiographic trends are a predictor of infield progression. 

## Materials and methods

Patient selection

This study was conducted under the approval of our institutional review board and represents a retrospective cohort study. Between May 2011 and May 2017, 580 patients with cerebral metastases underwent Gamma Knife (GK) SRS at our institution. Patients were excluded from the study if they (1) failed to have two post-GK treatment follow-up MRI scans, (2) underwent treatment for 10 or more cerebral metastases in a single or staged setting, and (3) underwent GK for only a post-surgical cavity. Patient with 10 or more metastases and staged radiation therapy were excluded to reduce confounding from variations in treatment dose and volume. A total of 406 patients were excluded from the study. Of the remaining 174 patients, a total of 579 cerebral metastases underwent GK radiosurgery and were included in the study analysis.

MRI scans and progression

As routine surveillance, patients generally undergo MRI scans every three months following SRS at our institution. Surveillance MRIs were retrospectively reviewed and lesion size was recorded in all three dimensions on T1 post-contrasted imaging. A lesion was interpreted as progression if new enhancement increased by at least 25% in two out of three dimensions. It was interpreted as decreased in size if new enhancement decreased by at least 25% in two out of three dimensions. All others were categorized as stable.

End points

Based on evaluation of progression, recurrences were categorized into three primary endpoints: (1) continued observation, (2) therapeutic intervention, and (3) withdrawal of care. Continued observation included those that demonstrated progression on a single MRI scan which were observed and evaluated on the next follow-up MRI scan for either continued progression or decrease/stabilization in size. Therapeutic intervention included those with continued progression requiring treatment with either craniotomy and surgical resection, repeat radiosurgery, or laser interstitial therapy. The decision for intervention was based on radiographic changes, patient symptomatology, and overall disease burden as evaluated by multidisciplinary board consisting of neurosurgeons, neuroradiologists, neurooncologists, and neuropathologists. The third primary endpoint, withdrawal of care, included patients who entered hospice care for one of various reasons, including overwhelming metastatic cancer burden.

Statistical analysis

Prism 7 software (GraphPad Software, Inc, San Diego CA) was used to complete the statistical data analyses. Continuous data are reported as means with standard error of the means. Categorical data are reported as frequencies and percentage. Statistical significance was defined as p<0.05. Volumes at time of surgical intervention were compared using Student’s t test.

## Results

Patient characteristics

Between May 2011 and May 2017, 174 patients harboring 579 cerebral metastases met inclusion criteria for this study (Table 1). The median length of follow-up was 10.5 months (1.9-48.7). Local control rate during the follow-up period was 84.2%. The mean tumor size at time of treatment was 1.2±2.5 cc.

**Table 1:** Patient demographics SRS, stereotactic radiosurgery

Patients (n)	174
Cerebral metastases treated w/ SRS (n)	579
Mean tumor volume (cc)	1.2±2.5
Median follow-up (months)	10.5 (1.9-48.7)
Local control rate	84.20%

**Figure 1:**
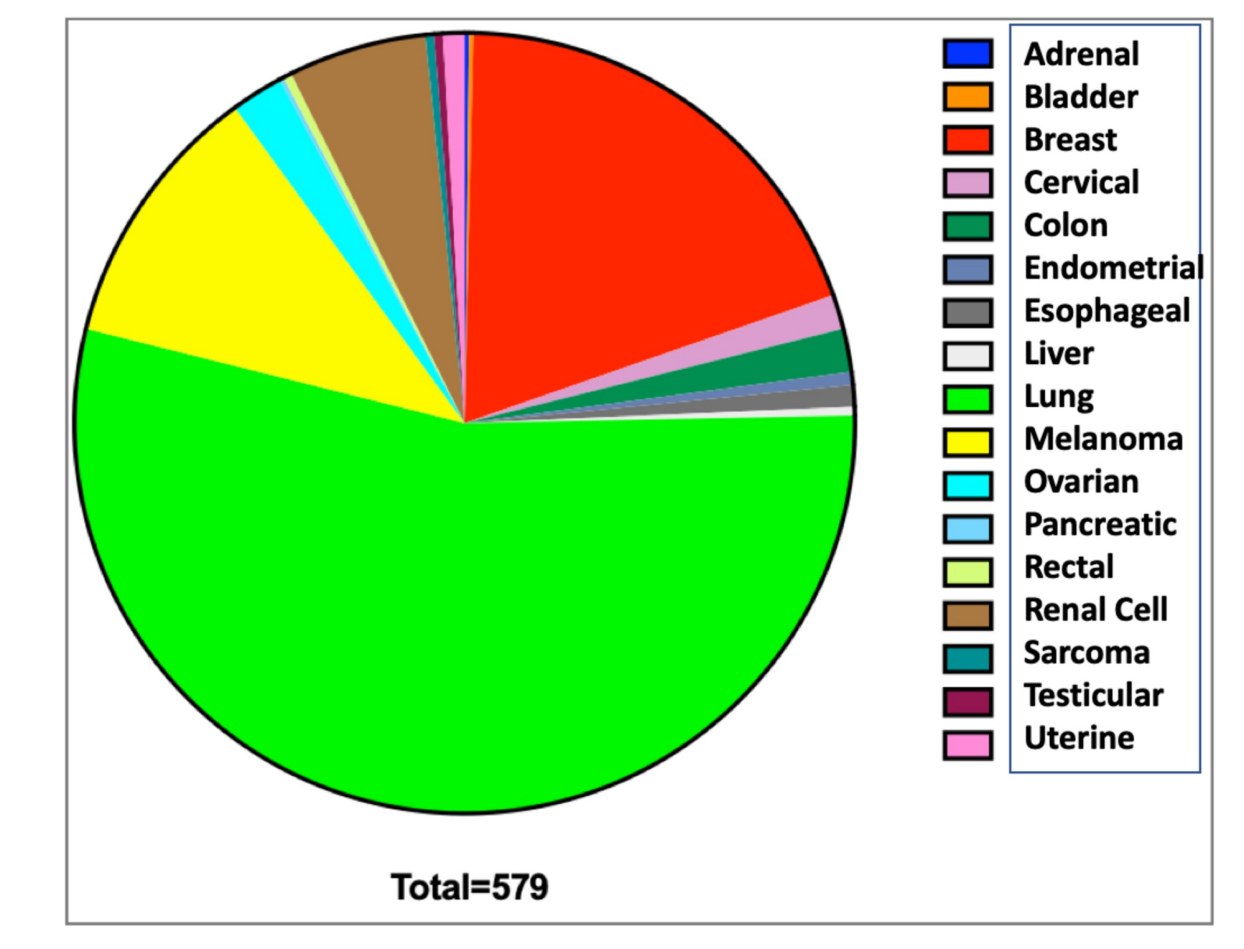
Cerebral metastases categorized by subtype

A variety of tumor pathologies were treated (Figure 1). The most common cerebral metastases treated were lung (52.0%), breast (18.7%), and melanoma (10.6%). 

Radiographic follow-up and outcome

**Figure 2:**
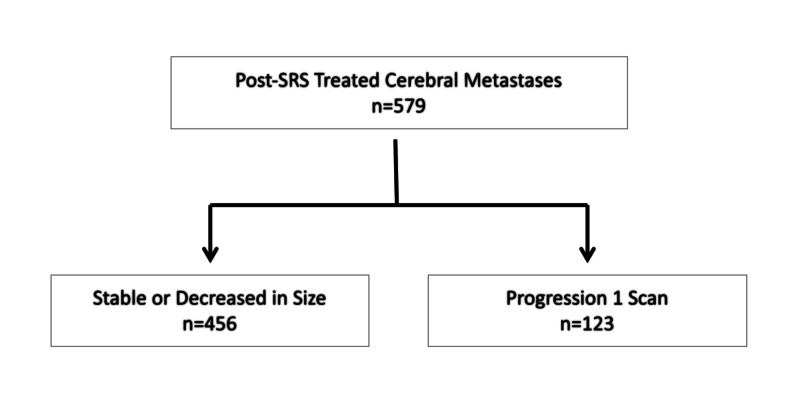
Cerebral metastases treated with GK SRS demonstrating infield progression GK SRS, Gamma Knife stereotactic radiosurgery

Of the 579 cerebral metastases treated with GK, 456 lesions remained stable or decreased in size during the course of radiographic follow-up (Figure 2). The remaining 123 lesions demonstrated progression on at least one surveillance MRI. The post-SRS treated cerebral metastases demonstrating progression were assessed with serial MRI and categorized into one of three primary endpoints (continued observation, treatment intervention, or withdrawal of care) at each time point (Table 2).

**Table 2:** Outcomes of radiological follow-up

	Progression 1 scan	Progression 2 scans	Progression 3 scans	Progression 4 scans	Progression 5 scans
	n=123	n=77	n=77	n=13	n=4
Continue to follow	103 (84%)	40 (52%)	13 (41%)	4 (35%)	0 (0%)
Stable/decrease on follow-up scan	26 (25%)	6 (15%)	0 (0%)	0 (0%)	0 (0%)
Continued progression	77 (75%)	34 (85%)	13 (100%)	4 (100%)	0 (0%)
Intervention	14 (20%)	28 (36%)	21 (62%)	9 (64%)	4 (100%)
Surgical resection	5 (4%)	4 (5%)	1 (3%)	0 (0%)	0 (0%)
Repeat radiosurgery	1 (0.8%)	10 (13%)	2 (6%)	5 (36%)	2 (50%)
Laser ablation	8 (6%)	14 (18%)	14 (41%)	4 (29%)	2 (50%)
Withdrawal of care	6 (6%)	9 (12%)	4 (12%)	0 (0%)	0 (0%)

Of the 123 recurrences demonstrating progression on one follow-up MRI scan, 103 (84%) were treated with continued observation, 14 (20%) required treatment intervention with either craniotomy and surgical resection, repeat radiosurgery, or laser ablation, and in six lesions patients underwent withdrawal of care due to overall disease burden. For those undergoing continued observation, on the next follow-up scan 25% of lesions demonstrated stable or decreased size, whereas 75% continued to progress. A total of 77 recurrences demonstrated progression in size on two consecutive MRI scans. In this group, 52% continued observation, 36% required treatment intervention, and 12% underwent withdraw of care. Of the 40 recurrences observed, 34 (85%) demonstrated continued progression on the following MRI scan and six (15%) stabilized or decreased in size. Thirty-four recurrences demonstrated progression on three consecutive MRI scans. A majority, 64%, required surgical intervention. Thirteen were treated with observation, and all of which demonstrated continued progression on their next follow-up scan. Of these remaining 13 lesions, four were continued to be observed and demonstrated progression on their fourth follow-up scan. All remaining lesions required surgical intervention by their fifth scan demonstrating progression.

Trends in infield progression on MRI

**Figure 3:**
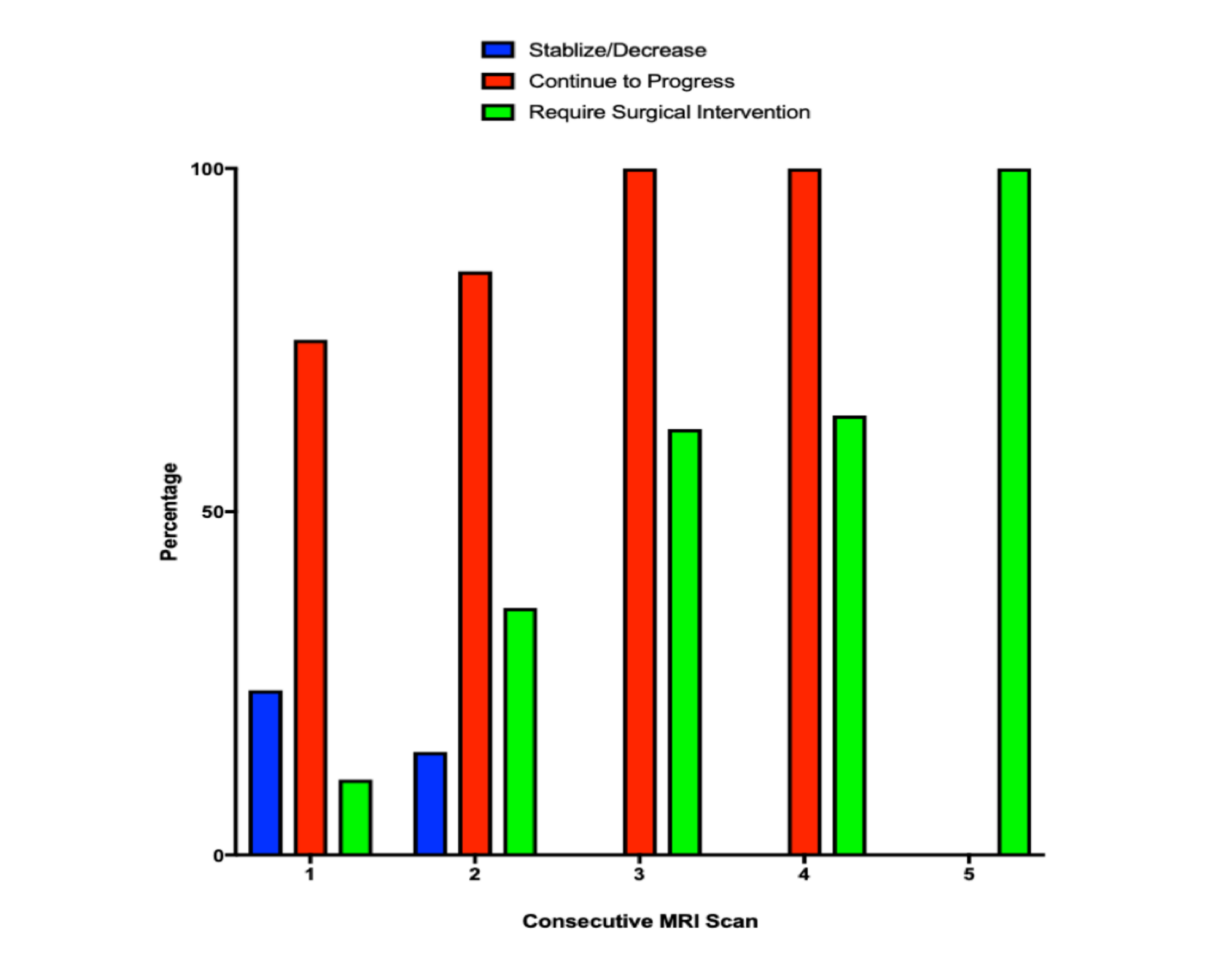
Trends in infield progression, stabilization, and requirement for surgical intervention The likelihood of infield progression stabilizing decreased from 25% after one MRI, 15% after two MRIs, to 0% after ≥3 MRIs. The likelihood of continued progression or need for intervention increased with the number of MRIs demonstrating progression.

Overall trends in infield progression, stabilization, and requirement for surgical intervention are demonstrated in Figure 3. For those demonstrating progression on one surveillance MRI, 25% will stabilize or decrease in size on the following surveillance MRI if treated with observation alone. For lesions demonstrating progression on two consecutive MRIs, the likelihood of stabilization or decrease in size decreased to 15%. All recurrences which demonstrated progression on three consecutive MRI scans would continue to progress if observed or eventually require surgical intervention. The need for surgical or radiosurgical intervention also increased with the number of MRI scans demonstrating continued progression. With progression on only one MRI scan, the rate of intervention was 20%. This rate was increased to 36% on two consecutive MRI scans demonstrating progression and 62% on three consecutive scans. By five consecutive MRI scans demonstrating progress, all lesions required intervention.

Volume at time of surgical intervention

**Figure 4:**
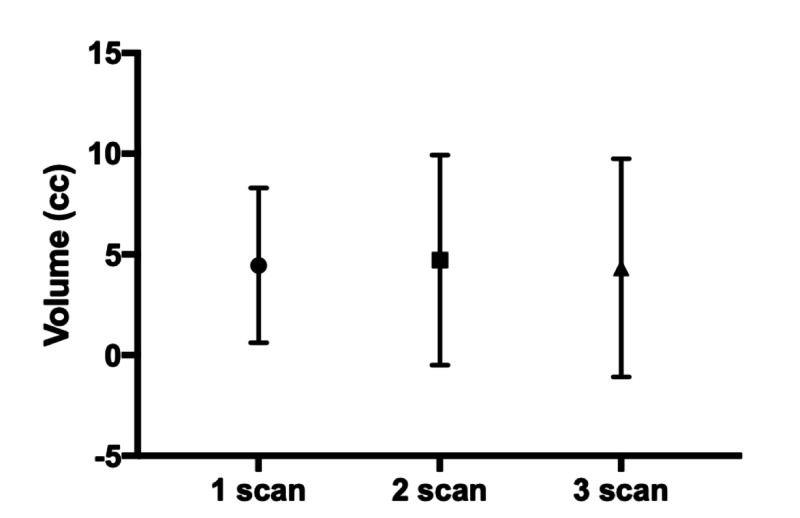
Lesion volume at time of intervention

**Figure 5:**
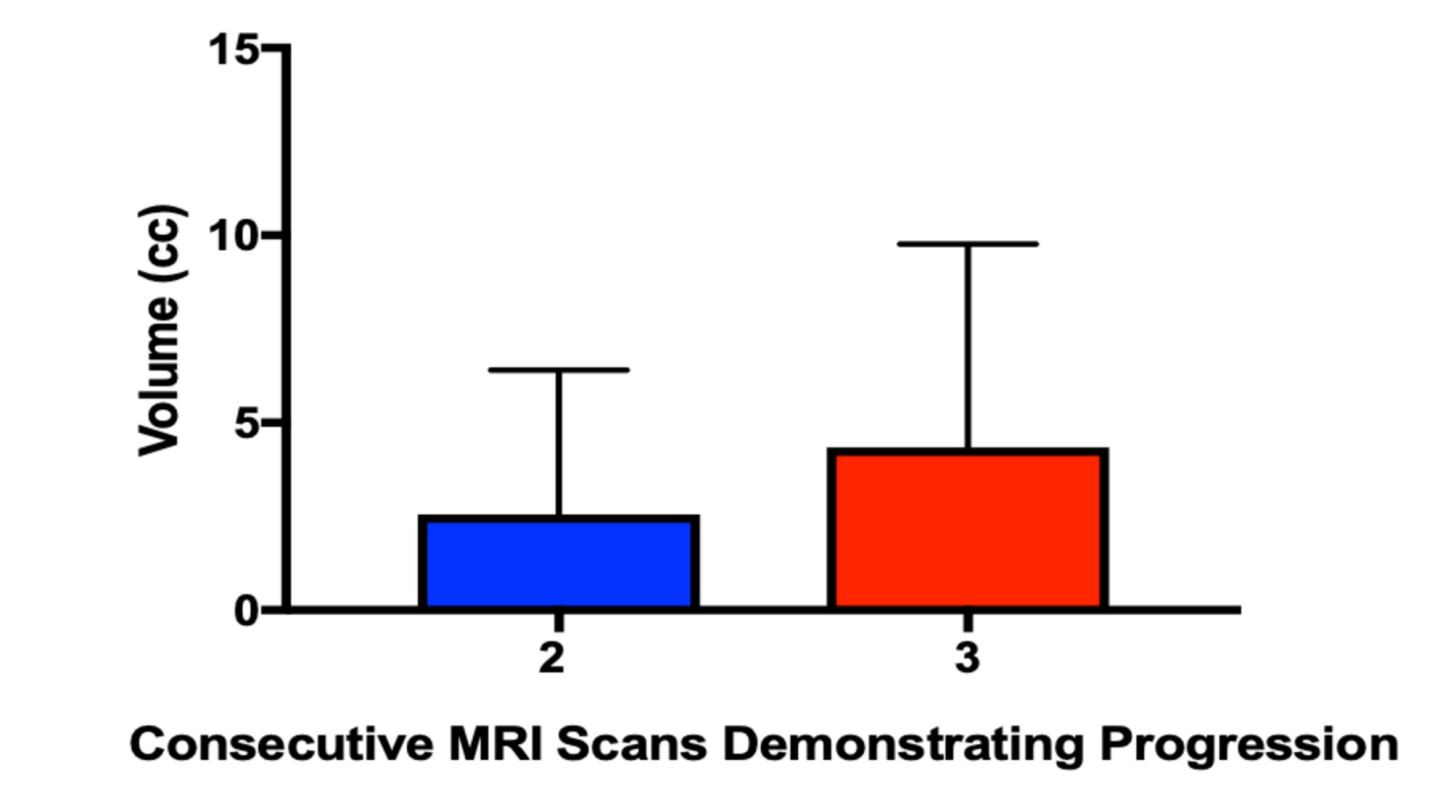
Lesion volume at time of intervention for recurrences which demonstrated progression on ≥3 MRIs Lesion volume at time of intervention for recurrences which demonstrated progression on ≥3 MRIs compared to volume if recurrence had been treated after the second MRI demonstrating progression. Intervention after two consecutive MRIs demonstrating growth allows lesions to be treated at smaller volume (2.6±3.8 cc vs 4.3±5.4 cc, p=0.03)

For recurrences demonstrating progression requiring surgical intervention, the volume was measured at the time of intervention (Figure 4). There was no statistical difference in overall volume between those operated on after the first (4.5±3.8 cc), second (4.7±5.7 cc), or third (4.3±5.4 cc) surveillance MRI (p>0.05). Since 100% of recurrences demonstrating progression on three or more surveillance MRIs would go on to require an intervention, we measured their size after two surveillance MRIs and compared it to the size after three scans. Intervention after two consecutive MRIs demonstrating growth allows recurrences to be treated at smaller volume (Figure 5, 2.6±3.8 cc vs 4.3±5.4 cc, p=0.03)

## Discussion

Brain metastases are the most common intracranial neoplasm, affecting 20%-40% of patients with metastatic cancer [16]. An aging population, advances in diagnostic imaging and surveillance, and improved cancer therapeutics resulting in increasing survival rates have contributed to the increasing incidence of brain metastases [17]. Subsequently, there has been an increasing trend in the use of SRS as an upfront treatment for brain metastases [18]. SRS is a safe and effective therapy for the treatment of brain metastases [3,7,19]. SRS has demonstrated equivalent survival rates and less cognitive side effects compared to whole brain radiation [20-22]. While typically utilized for treatment of one to three brain metastases less than 3 cm in size, studies have shown SRS to be effective for the treatment of increasing disease burden, both in size and number of cerebral metastases [23-25]. Despite being an effective therapy, treatment failure following SRS can occur in up to 30% of treated tumors [2,3,5,6]. Clinical decision making in regards to recurrence treatment can be challenging.

Recurrence following SRS can represent recurrent tumor or radiation necrosis [8]. Clinical decision making in regards of recurrence treatment is often based on distinguishing between the two [13]. A fundamental flaw in this clinical philosophy is the belief that if the recurrent enhancement represents radiation vasculitis or necrosis, it will ultimately stabilize without the need for treatment, whereas recurrent tumor would require additional intervention with either surgery or radiation [26]. However, radiation necrosis can demonstrate continued progression, and predicting which patients will demonstrate progression has proven challenging for any particular patient. Diagnosing radiation necrosis vs recurrent metastasis can be challenging based on radiographic modalities alone [27]. Biopsies of these lesions can display mixed results, with areas of both recurrent tumor and necrosis on pathological specimens [10]. So are we are really asking the right question to guide treatment, “radiation necrosis vs recurrent metastasis”? Perhaps the right question to ask is whether the recurrent lesion is “progressive”? At our institution, we treat radiation necrosis and recurrent tumor following SRS based on the idea that it has become irreversibly progressive, irrespective of the underlying pathophysiology. We have termed the phrase progressive enhancing inflammatory reactions, or PEIRs, a radiographic diagnosis based on continued and increasing enhancement following SRS [14].

In order to better assess progression following GK SRS, an understanding of post-GK volumetric response is necessary. In the immediate post-treatment period, less than 30 days, tumors can display a dynamic volumetric response to GK treatment, with some lesions increasing in size, some lesions decreasing in size, and others remaining stable [3]. Prior studies have looked at risk factors associated with metastasis which fail SRS and include factors such as age, number of brain metastases, tumor volume, melanoma history, and progressive systemic disease [28]. However, once a treated tumor demonstrates infield progression, it is difficult to predict which will demonstrate continued progression or which will potentially stabilize or even regress. Thus, the timing of intervention remains unclear, although as these grow and become symptomatic requiring steroids, the treatment becomes more challenging. 

The purpose of this study was to retrospectively review radiographic trends in infield progression following SRS for brain metastasis to understand what happens after the first evidence of growth. We found that for recurrences demonstrating progression on one MRI scan, 25% will exhibit stabilization or decrease in size on their following surveillance scan. For those which demonstrated continued progression on their second consecutive MRI, only 15% of lesions stabilized or regressed on their following scan. Once progression occurs over three consecutive MRIs, no further stabilization or regression was evident with further observation. Since 91% of recurrences will either require intervention or demonstrate continued progression with observation once progression is documented on two consecutive surveillance MRIs, these should be considered GK failures and considered for further additional interventions.

Symptomatic brain metastases can have a profound impact on patient quality of life and heavy economic burden on the healthcare system. Early detection and intervention can lead to better clinical outcomes and healthcare savings [29]. Similarly, there are several benefits to early detection of infield progression leading to intervention when lesion size is small and patients are less symptomatic. For patients requiring craniotomy and surgical resection, smaller tumor burden is associated with reduced risk of post-operative complications [11]. For patients undergoing repeat SRS for infield recurrence, larger retreatment volumes are associated with increased likelihood of poor neurological outcomes [12]. LITT demonstrates improved clinical efficacy when infield recurrences are treated at smaller volumes [30]. Additionally, patients with larger lesions requiring high-dose steroids were found to be more likely to encounter a post-ablation complication [14]. In our patient cohort, the average size of recurrence at the time of treatment was approximately 4 cc. There was no significant difference in size whether the lesion was treated after the first, second, or third surveillance MRI. However, since 100% demonstrating progression on three or more consecutive surveillance MRIs required treatment, we measured recurrence size at time of intervention compared to size at the time of progression on two consecutive MRIs. If these recurrences had been treated at the time of their second MRI demonstrating progression, treatment volume would have been significantly smaller, 2.6±3.8 cc vs 4.3±5.4 cc (p=0.03). Treatment at a smaller size is potentially easier on the patient but carries with it an approximately 10% rate of treating false positives.

This study has a few limitations. First, at our institution we do not perform biopsies prior to intervention once we make the diagnosis of infield progression. While we do not know the true biology of infield recurrence we are treating, our decision for treatment is on the basis of progression, not tissue diagnosis. Many of these are treated with LITT, and the biopsy at the time of ablation introduces the risk of signal artifact during the procedure. Secondly, our institution is a high-volume LITT center. Approximately 58% of infield progression requiring intervention was treated with LITT in our study. We have a tendency to favor LITT when lesions are smaller before patients become symptomatic due to better clinical efficacy and less risk of adverse complications. Thus, we may be creating a potential bias toward earlier treatment of smaller lesions. It is quite possible that some of the treated recurrences may have stabilized if they were observed for longer. Additionally, it is important to reinforce that we defined progression specifically as growth by about 25%, and if this definition is changed, these numbers may look different. We acknowledge our patient cohort consists of a heterogeneous population of primary tumor pathologies and this study does not take into account systemic disease status. Overall disease status and systemic treatments may play a role in post-SRS progression. Larger multicenter studies with different treatment preferences are needed. 

## Conclusions

Approximately one-fourth of infield recurrence demonstrating progression on the first surveillance MRI will stabilize or regress. Recurrences demonstrating infield progression on two consecutive MRI scans should be considered treatment failures. Early interventions before tumor volume increases in size or patients require high-dose steroids maybe beneficial. Prospective studies may answer this question in a more definitive manner.
